# National stakeholder preferences for next-generation rotavirus vaccines: Results from a six-country study

**DOI:** 10.1016/j.vaccine.2021.11.009

**Published:** 2022-01-21

**Authors:** Jessica Price, Jessica Mooney, Carolyn Bain, John Tanko Bawa, Nikki Gurley, Amresh Kumar, Guwani Liyanage, Rouden Esau Mkisi, Chris Odero, Karim Seck, Evan Simpson, William P. Hausdorff

**Affiliations:** a PATH, Seattle, 2201 Westlake Ave, Seattle, WA 98121, USA; b PATH, Ghana, PMB CT 307 Cantonments Accra, Ghana; c PATH, India, 15th Floor, Dr. Gopal Das Bhawan 28, Barakhamba Road, Connaught Place, New Delhi 110001, India; d Department of Pediatrics, Faculty of Medical Sciences, University of Sri Jayewardenepura, Sri Lanka; e PATH, Malawi, Private Bag B381 Capital City, Lilongwe 3, Malawi; f PATH, Kenya, ACS Plaza, 4th Floor Lenana and Galana Road, P.O. Box 76634-00508, Nairobi, Kenya; g PATH Senegal Consultant, Fann Résidence Rue Saint John Perse X F Dakar, Senegal; h PATH, Washington, DC, 455 Massachusetts Ave. NW, Suite 1000, Washington, DC 20001, USA; i Université Libre de Bruxelles, Brussels, Belgium

**Keywords:** Rotavirus, Rotavirus vaccines, Next-generation rotavirus vaccines, Value proposition, New vaccine introduction, Vaccine decision-making

## Abstract

**Background:**

Currently available live, oral rotavirus vaccines (LORVs) have significantly reduced severe rotavirus hospitalizations and deaths worldwide. However, LORVs are not as effective in low- and middle-income countries (LMIC) where rotavirus disease burden is highest. Next-generation rotavirus vaccine (NGRV) candidates in development may have a greater public health impact where they are needed most. The feasibility and acceptability of possible new rotavirus vaccines were explored as part of a larger public health value proposition for injectable NGRVs in LMICs.

**Objective:**

To assess national stakeholder preferences for currently available LORVs and hypothetical NGRVs and understand rationales and drivers for stated preferences.

**Methods:**

Interviews were conducted with 71 national stakeholders who influence vaccine policy and national programming. Stakeholders from Ghana, Kenya, Malawi, Peru, Senegal, and Sri Lanka were interviewed using a mixed-method guide. Vaccine preferences were elicited on seven vaccine comparisons involving LORVs and hypothetical NGRVs based on information presented comparing the vaccines’ attributes. Reasons for vaccine preference were elicited in open-ended questions, and the qualitative data were analyzed on key preference drivers.

**Results:**

Nearly half of the national stakeholders interviewed preferred a highly effective standalone, injectable NGRV over current LORVs. When presented as having similar efficacy to the LORV, however, very few stakeholders preferred the injectable NGRV, even at substantially lower cost. Similarly, a highly effective standalone injectable NGRV was generally not favored over an equally effective oral NGRV following a neonatal-infant schedule, despite higher cost of the neonatal option. An NGRV-DTP-containing combination vaccine was strongly preferred over all other options, whether delivered alone with efficacy similar to current LORVs or co-administered alongside an LORV (LORV + NGRV-DTP) to increase efficacy.

**Conclusion:**

Results from these national stakeholder interviews provide valuable insights to inform ongoing and future NGRV research and development.

## Highlights

• An effective and affordable injectable rotavirus vaccine may be attractive to LMICs.
• Co-administering oral and injectable vaccines is acceptable to many stakeholders.
• Oral vaccine with a birth dose is favored over a higher cost, standalone injectable.
• Providing rotavirus vaccine in a DTP combination is the most preferred option.

## 1 Introduction

### 1.1 Rotavirus burden and the role of vaccines

Despite availability of rotavirus vaccines recommended for use in all countries by the World Health Organization (WHO) in 2009 [1], rotavirus burden remains high globally. Rotavirus accounts for a quarter to one-third of under-five mortality due to diarrhea, resulting in 120,000 to 220,000 deaths annually [2], [3]. While all children are susceptible to rotavirus infection, the majority of deaths occur in low- and middle-income countries (LMICs), more than half in sub-Saharan Africa [3]. Improvements in hygiene, water quality, and sanitation that prevent spread of many bacteria and parasites do not adequately prevent rotavirus infections [4], [5]. Vaccines are the best way to protect children from mortality and morbidity caused by severe rotavirus diarrhea [6].

Six live, oral rotavirus vaccines (LORVs) are currently prequalified by the WHO for procurement by United Nations agencies and Gavi, the Vaccine Alliance for use in LMICs [7], [8], [9], [10], [11]. Gavi began funding rotavirus vaccine introductions in 2007. As of August 2021, 113 countries had introduced LORV, reaching an estimated 59% of vaccination-age children globally [12]. Seventy-four percent of the highest-burden countries in sub-Saharan Africa have introduced LORV [13]. However, uptake in some regions and high burden countries has been slow [14] stemming from low perceived need for a rotavirus vaccine coupled with perceived and actual vaccine cost barriers, particularly in middle-income countries [15], [16].

While LORV introductions have reduced the burden of under-five diarrheal disease [17], currently licensed products show a marked disparity in protection against severe rotavirus gastroenteritis based on the socioeconomic status of the vaccinated population [7], [11], [18]. Whereas studies in infants and toddlers in higher-income countries report 80–95% protection against severe disease, the same vaccines have only demonstrated 50–60% effectiveness in LMICs. The reasons behind diminished LORV protection in LMICs remain unclear [19]. Additionally, in some settings, LORVs have been associated with a slightly elevated risk of intussusception [20].

Next-generation rotavirus vaccines (NGRVs) may address these efficacy and safety issues. NGRVs include parenteral and oral candidates [21], some in phase II or III clinical trials (see ClinicalTrials.gov NCT03483116 and NCT04010448). Each NGRV presents unique operational features and costs which could affect their appeal to LMICs. As part of a larger PATH public health value proposition for NGRVs, we conducted a feasibility and acceptability (F&A) study to assess the attractiveness of different NGRVs among key LMIC stakeholders.

### 1.2 Public health value proposition for NGRVs

Taking into account various elements of value propositions, full value of vaccine assessments and investment cases [22], [23], and specific questions surrounding the value of NGRVs, PATH’s value proposition had three main components: (1) a mixed-method F&A study, the subject of this paper, to elicit stakeholder preferences for and views on hypothetical NGRVs; (2) economic modeling to establish scenarios in which hypothetical NGRVs are projected to hold public health or economic benefits over currently available LORVs; and (3) demand forecasting to quantify potential market sizes for different NGRVs.

The F&A elicited LMIC stakeholder preferences for current LORVs versus hypothetical NGRVs featuring different presentations, efficacy, schedule, dose regimens, cold chain volume requirements, and cost. Specifically, we sought to understand which attributes in what circumstances are most valued. For instance, would an injectable NGRV with similar efficacy to LORVs be valued? If so, in what circumstances? Which vaccine attributes in what contexts would outweigh the advantages of a lower-cost product?

We focused on two hypothetical NGRVs to answer such questions. The first is a parenteral vaccine, denoted as ***i***NGRV, envisioned as a three-dose presentation administered via intramuscular injection. iNGRV is modeled after the trivalent P2-VP8 subunit vaccine candidate [24]. Trivalent P2-VP8 may offer enhanced efficacy compared to LORVs, would eliminate risk of intussusception, and has the potential for combinability with currently available parenteral vaccines given along the infant schedule. The second hypothetical NGRV is an oral vaccine, denoted by ***o***NGRV, with an initial birth dose followed by two doses given in the routine infant schedule. oNGRV is modeled after the human neonatal rotavirus vaccine candidate RV3-BB [25]. RV3-BB may have higher efficacy than current LORVs, while reducing risk of intussusception and providing infants early protection against rotavirus infection [21], [25]. Both trivalent P2-VP8 and RV3-BB are in late-stage clinical development.

Much of the current NGRV research and development is underpinned by four prevailing assumptions:

1. The highest priority for development of any new rotavirus vaccine should be demonstration of higher efficacy (rather than, for example, greater convenience in presentation or storage) [21].
2. Any new standalone NGRV requiring multiple injections, regardless of efficacy, would likely be rejected [26].
3. The co-administration of an LORV with a moderately effective iNGRV to achieve higher efficacy (see ClinicalTrial.gov, NCT04344054) would likely be considered too complicated and/or costly to adopt.
4. A high-efficacy oNGRV following a neonatal-infant schedule would likely be preferred over all other options, given its oral route of administration and potential to increase vaccination coverage, to provide protection at an earlier age, and to minimize risk of intussusception [21], [25].

Rarely consulted in advance of vaccine development, LMIC stakeholder views about hypothetical NGRVs could help to assess the validity of these assumptions.

## 2 Material and methods

Data were collected from three study groups. We first conducted informational interviews with international rotavirus vaccine experts to elicit their perspectives on priorities and issues. Interviews with national stakeholders and healthcare providers were then conducted to identify vaccine preferences and programmatic challenges. The methods and findings from health provider interviews are reported elsewhere (J. Mooney, unpublished results). This paper presents results from the national stakeholder sample.

### 2.1 Sample

Six countries were purposively selected to represent different geographic regions, socio-economic status, and eligibility for Gavi co-financing (Table 1). Malawi and Senegal, both in Gavi’s ‘initial self-financing’ phase, contribute a flat amount of US$0.20 per vaccine dose. Ghana and Kenya, in the ‘preparatory transition’ phase, contribute more at a 15% price increase per year [27]. Peru and Sri Lanka are not eligible for Gavi support. Five of the countries had introduced an LORV within the last 7–12 years. Sri Lanka has not yet introduced an LORV.

**Table 1:** Countries Selected, Gavi Status, and LORV Introduction Date.

Country	Gavi Status (2019)	Date of LORV^1^ Introduction
Ghana	Preparatory transition phase	April 2012
Kenya	Preparatory transition phase	July 2014
Malawi	Initial self-financing	October 2012
Peru	Not eligible	January 2009
Senegal	Initial self-financing	November 2014
Sri Lanka	Not eligible	Not applicable


^1^Live oral rotavirus vaccine.

Individuals involved in vaccine policy and decision making, or who play key functional, technical, or advisory roles, were invited to be interviewed, with a goal to recruit 10–15 stakeholders per country. We did not recruit individuals from Ministries of Finance.

### 2.2 Vaccine attributes

Interviews focused on seven vaccine comparisons involving one of three LORVs and six different NGRVs. Country-specific visual aids containing information about known (LORVs) or assumed (hypothetical NGRVs) attributes for each vaccine in the comparison were displayed to the interviewee. Table 2 details assumptions used to prepare the visual aids. Supplement 1 shows information used in country-specific visual aids. Pricing information for the LORVs was obtained from publicly available sources including the Gavi detailed product profiles [28] and the WHO vaccine purchase database [29]. As no NGRV is yet on the market, hypothetical prices for iNGRV and oNGRV were informed by personal communications with vaccine developers and other experts. Prices displayed in the visual aids reflected assumed Gavi co-financing status in 2025.

**Table 2:** Attributes of Vaccines Used in the Comparisons.

Existing & Hypothetical Vaccines		Assumed Efficacy	Presentation	Route of administration & dosage	Schedule & number of doses	Cold chain volume/FIC (cm3)^4^	Cost/FIC (2025) Relative to Comparator LORV^5–7^
Comparator LORV	ROTARIX^1^	Varies by country (50–80%)^3^	Plastic strip of 5 tubes	Oral; 1.5 mL	2 doses at 6 & 10 weeks	23.6	100% (reference price)
	ROTAVAC^1^		5-dose vial	Oral; 5 mL (5 drops)	3 doses at 6, 10, 14 weeks	12.6	
	ROTASIIL^1^		2-dose lyophilized vial plus diluent	Oral; 2.5 mL	3 doses at 6, 10, 14 weeks	31.6	
NGRV	iNGRV-H^2^	80%	2-dose vial without preservative	Injectable; .5mL	3 doses at 6, 10, 14 weeks	46.2	*Initial self-financing*: 0% decrease^8a^ *Preparatory transition*: 25–50% decrease^8b^ *Non-Gavi eligible:* 25–50%, decrease^8c^
	iNGRV-M^2^	=comparator LORV	2-dose vial without preservative	Injectable; .5mL	3 doses at 6, 10, 14 weeks	46.2	
	iNGRV-DTP (iNGRV-DTP-Hib-HepB)^2^	=comparator LORV	iNGRV-DTP-Hib-HepB containing combination	No additional injections	iNGRV-DTP given at 6, 10, 14 weeks	No additional cold chain	*Initial self-financing*: 100% decrease^8a^ *Preparatory transition*: 60–75% decrease^8b^ *Non-Gavi eligible*: 60–75% decrease^8^^c^
	Co-admin 1 (LORV + iNGRV-M)^2^	80%	Comparator LORV + iNGRV-M	Comparator LORV + iNGRV-DTP	Comparator LORV schedule + injectable at 6, 10, 14 weeks	Comparator LORV volume + 46.2	*Initial self-financing*: 100% increase^8a^ *Preparatory transition*: 50–75% increase^8b^ *Non-Gavi eligible*: 50–75%% increase^8c^
	Co-admin 2 (LORV + iNGRV-DTP)^2^	80%	Comparator LORV + iNGRV-DTP	Comparator LORV + Injectable; .5mL	Comparator LORV + iNGRV-containing DTP at 6, 10, 14 weeks	Comparator LORV volume	*Initial self-financing*: 0% increase^8a^ *Preparatory transition*: 25–40% increase^8b^ *Non-Gavi eligible*: 25–40% increase^8c^
	oNGRV^2^	80%	Plastic strip of 5 tubes	Oral; 1 mL	3 doses: neonatal, 6 and 10 weeks	23.6	*Initial self-financing*: 0% change^8a^ *Preparatory transition*: 20% increase to 25% decrease^8b^ *Non-Gavi eligible*: 20% increase to 25% decrease^8c^

^1^Currently available live oral rotavirus vaccines (LORVs) used as comparators.

^2^Next generation rotavirus vaccines (NGRV) under development, including injectable (iNGRV) and oral (oNGRV) candidates.

^3^LORV efficacy and waning assumptions were based on pooled data from published randomized controlled trials of rotavirus vaccines [2]. They were used to estimate country-specific deaths and hospitalizations averted over a 10-year period.

^4^Cold chain requirement assumptions for the parenteral NGRVs were unintentionally based on secondary rather than primary packaging sizes and thus were substantially larger than they would actually be.

^5^Varies by country and comparator LORV.

^6^For Gavi eligible countries (Gavi), prices informed by Gavi rotavirus vaccine product profiles and estimates of co-financing price fractions. For Gavi non-eligible countries (Non-Gavi), prices estimated based on the WHO V3P database. https://www.who.int/teams/immunization-vaccines-and-biologicals/vaccine-access/mi4a/mi4a-vaccine-purchase-data.

^7^Cost assumptions for iNGRV provided by Stan Cryz, Director, Non-replicating Rotavirus Vaccine Program. PATH, Washington DC. Assumed vaccine costs were then adjusted using the methods described above in footnote 4.

^8a^Malawi and Senegal.

^8b^Ghana and Kenya.

^8c^Peru and Sri Lanka.

Throughout this paper, iNGRV***-H*** signifies a standalone injectable vaccine assumed to have **higher** efficacy than current LORVs. iNGRV***-M*** signifies a standalone injectable assumed to have **medium** efficacy, similar to current LORVs. iNGRV-**DTP** denotes an NGRV-DTP-containing (e.g., DTP HepB-Hib) combination vaccine. Co-admin 1 refers to the co-administration of an LORV with iNGRV-M. Co-admin 2 refers to the co-administration of an LORV with iNGRV-DTP.

### 2.3 Interviews

Individual in-person interviews followed a semi-structured guide that included fixed-choice and open-ended questions (Supplement 2). Lasting 40–45 min, the interview covered stakeholder roles, perceptions about rotavirus and LORV introduction, and vaccine preferences. Vaccine comparison questions proceeded in three steps (Table 3) to: (1) clarify cross-cutting assumptions about all vaccines in the comparisons; (2) identify a comparator LORV to be used in subsequent comparisons with NGRVs; and (3) elicit vaccine preferences comparing two vaccines at a time. Stakeholders were asked to review information shown in the visual aid and, when ready, to state their preference and explain reasons for their choice.

**Table 3:** Vaccine Comparisons.

Step 1: Stakeholders asked to assume that all vaccines in the comparisons: • Are WHO prequalified • Are supported by Gavi co-financing for eligible countries • Have a shelf-life of 24 months at 2–8 °C • Have comparably good safety profiles			
Step 2: Stakeholders select a comparator LORV			
Step 3: Stakeholders indicate vaccine preference on seven core comparisons (C1-C7)			

Key question 1: *Would a standalone iNGRV be a preferred alternative to LORV if it averted more child deaths and hospitalizations, was less costly to procure, or both?*	C1	LORV^1^	iNGRV-H^3^
	C2	LORV^1^	iNGRV-M^3^

Key question 2: *If an iNGRV is not found to be substantially more efficacious than LORVs, are there formulations in which it would be preferable to LORVs?*	C3	LORV^1^	Co-admin 1^4^
	C4	LORV^1^	Co-admin 2^4^
	C5	LORV^1^	iNGRV-DTP^5^

Key question 3: *What are stakeholder preferences for and views about an LORV with an initial neonatal dose (oNGRV) compared to equally efficacious iNGRV options?*	C6	oNGRV^2^	iNGRV-H^3^
	C7	oNGRV^2^	iNGRV-DTP^5^

^1^Live oral rotavirus vaccine.

^2^Oral next generation rotavirus vaccine (oNGRV).

^3^Standalone injectable next generation rotavirus vaccine (iNGRV) with high (-H) and moderate (-M) efficacy.

^4^Co-admin 1 = LORV + iNGRV-M; Co-admin 2 = LORV + iNGRV-DTP.

^5^iNGRV-DTP-Hib-HepB containing combination.

Comparisons 1–5 (C1-C5) included the comparator LORV versus iNGRVs assumed to have different price points, presentations, and efficacy compared to LORV. C1-C2 assumed the standalone iNGRVs had the same cost, but different efficacy. C3-C4 involved mixed schedules, where LORV and iNGRV-M are both given; these comparisons were included given that high efficacy may be achievable only through co-administration of two vaccines working through different immune mechanisms. C5 included iNGRV-DTP, intended principally to explore attractiveness of a combination vaccine if it confers similar protection to LORV. C6-C7 involved comparisons between two hypothetical NGRVs assumed to have similar efficacy. C6 compared oNGRV to standalone iNGRV and C7 compared oNGRV to iNGRV-DTP.

### 2.4 Data processing

iPads were used to record the interviews and collect data from fixed-choice questions using Research Electronic Data Capture (REDCap) tools [30]. Data captured through REDCap were automatically uploaded to datafiles. Audio-recordings were translated into English, if needed, and transcribed in full. Transcripts were coded using NVivo 12 Pro [31]. Where discrepancies between the transcript and quantitative data were found, the quantitative datafile was corrected to align with responses recorded in the transcript.

### 2.5 Data analysis

Textual and quantitative data were analyzed independently and together applying cross-over mixed analytic approaches [32]. Frequencies distributions on quantitative data were determined. Textual data were coded following an initial broad coding scheme that was iteratively refined through team-based, inductive coding [33], [34]. Select codes/categories were reduced to numeric variables [35] to reveal thematic patterns, to discern within-category diversity, and to merge with quantitative data for cross-over mixed analysis. Consensus-based coding/categorizing was done by two primary coders (JP and JM), with intermittent verification by study investigators.

Preference patterns: Within-case analysis was conducted on vaccine preferences to identify all unique preference pathways from C1 to C7. Across-case analysis involved visualizing preferences on all comparisons from all individuals to apprehend the overall pattern in the sample.

Preference drivers: On each comparison, replies to the question, *Why did you select vaccine X?* were coded to identify preference drivers. Driver codes were grouped into successively broader categories, eventually assigning all codes to one or more main drivers, including: IMPACT: references to vaccine efficacy and coverage. COST: references on to product cost per fully immunized child, Gavi co-financing, and long-term sustainability; references to operational costs (e.g., additional training) were coded to FEASIBILITY. FEASIBILITY: references to operational issues to store, transport, and administer the vaccine according to the schedule. ACCEPTABILITY: references to potential client or provider reluctance to receive or deliver the vaccine. When not associated with a specific operational concern (e.g., cold chain required), remarks about preferring to “avoid” new injections were also coded to ACCEPTABILITY.

Key questions: Cross-over mixed analysis was structured around three questions relevant to NGRV research and development:

1. Would a standalone iNGRV be a preferred alternative to LORV if it averted more child deaths and hospitalizations, was less costly to procure, or both?
2. If an iNGRV is not found to be substantially more efficacious than LORVs, are there formulations in which it would be preferable to LORVs?
3. What are stakeholder preferences for and views about an LORV with an initial neonatal dose (oNGRV) compared to equally efficacious iNGRV options?

Related to each of these key questions, we present frequency distributions of stakeholder preferences in tandem with qualitative findings describing reasons for their selections.

### 2.6 Ethics

Ethical approvals were obtained from PATH’s Research Ethics Committee, the Ghana Health Service Ethics Review Committee, the Kenyatta National Hospital-University of Nairobi Research Ethics Committee, Malawi’s National Health Sciences Research Committee, Peru’s Comité lnstitucional de Bioética, Senegal’s Comité National d'Ethique pour la Recherche en Santé, and in Sri Lanka approval was obtained from a SIDCER (Strategic Initiative for Developing Capacity in Ethical Review) recognized ERC, Faculty of Medical Sciences, University of Sri Jayewardenepura. Written informed consent was obtained from all participants.

## 3 Results

Between 8 and 14 stakeholders in each country agreed to be interviewed for a total sample of 71 individuals. Five stakeholders from Sri Lanka and one from Kenya declined to be interviewed. More than half of the stakeholders were current members of National Immunization Technical Advisory Groups (NITAGs) or equivalent bodies (Table 4).

**Table 4:** National Stakeholder Background by Country.*

Country	Total	Member of National Advisory Group	Ministry of Health Manager or Staff	Technical Assistance Agency	Clinician
Ghana	9	7	4	3	2
Kenya	14	9	8	6	0
Malawi	13	10	5	5	3
Peru	14	7	3	2	9
Senegal	8	8	4	2	2
Sri Lanka	13	1	0	0	13
***Total***	***71***	***42***	***24***	***18***	***29***

* Categories not mutually exclusive.

All stakeholders from Africa described rotavirus as “moderately” or “very” serious in their countries, most indicating that the introduction of LORV “has helped but more needs to be done.” In Peru, half of the stakeholders described rotavirus as “not very serious,” with several attributing this to LORV introduction. No individuals from Sri Lanka, where LORV has yet to be introduced, thought rotavirus was a serious problem, describing it as “seldom seen,” “not a top cause of mortality or morbidity,” or already well managed by other interventions. These findings generally reflect differing rotavirus burdens in stakeholders’ respective countries.

### 3.1 Vaccine preferences

**Fig. 1:**
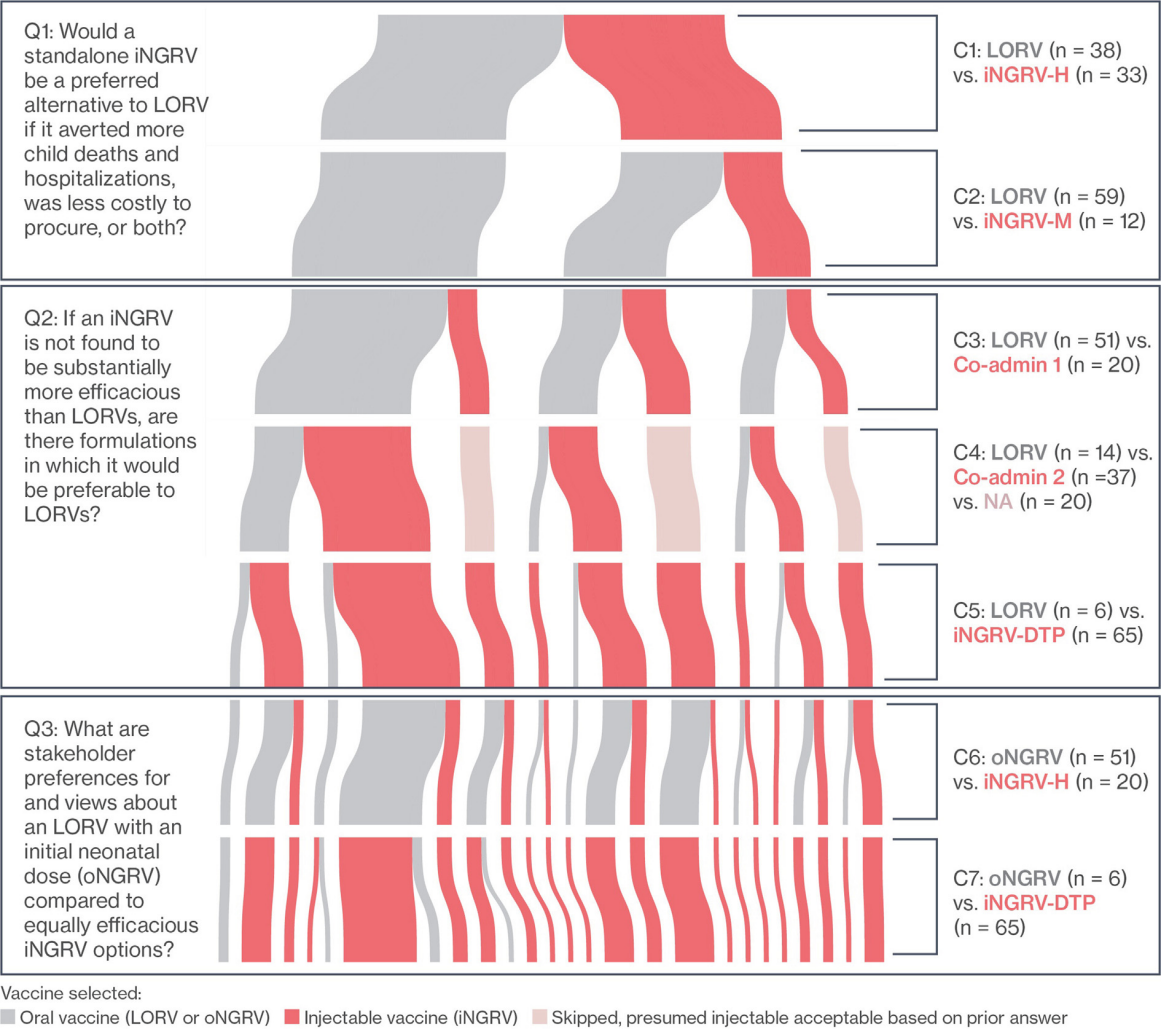
Stated Preferences for Vaccine Options C1-C7 by Oral and Injectable Mode of Delivery. LORV = live oral rotavirus vaccine; iNGRV-H = standalone injectable next generation rotavirus vaccine with high efficacy; iNGRV-M = standalone NGRV with moderate efficacy; Co-admin 1 = LORV+iNGRV-M; Co-admin 2 = LORV+iNGRV-DTP; iNGRV-DTP = iNGRV-DTP-Hib-HepB containing combination; oNGRV = Oral NGRV following neonatal-infant schedule.

Fig. 1 displays findings on stakeholder preferences for C1-C7. Moving down the figure, changes in color and line thickness show how preferences shift as vaccine attributes change from one comparison to next. The gray lines indicate preference for oral vaccine options – LORV (C1-C5) or oNGRV (C6-C7) – and the red/pink lines indicate preference for iNGRV options. Each individual’s preference in a comparison is linked to selections in previous and subsequent comparisons. Thickness of the lines is proportionate to the number of individuals who selected the same options in the present and all previous comparisons. Thin lines in C7, for example, indicate that only one or few individuals made all of the same selections from C1 to C7; thicker lines indicate several individuals selected the same options in all seven comparisons.

### 3.2 Unique preference pathways

Within case analysis found 25 unique preference pathways, displayed as 25 lines of varying thickness at C7 in Fig. 1. The most frequent pathway was found in 15 (21%) individuals and the five most frequent pathways in over half (54%) of the sample (Table 5). Two stakeholders selected the oral option (LORV or oNGRV) across all seven comparisons (outer-left, gray-colored line from C1-C7 in Fig. 1), while 25 others selected the oral option or iNGRV-DTP across all seven comparisons. Twelve of the stakeholders selected both standalone iNGRV-H and iNGRV-M, four among them preferring the iNGRV option across all seven comparisons (outer-right, red line from C1-C7 in Fig. 1).

**Table 5:** Unique Preference Pathways.

	Preference by Comparison							Stakeholders	
	C1	C2	C3	C4	C5	C6	C7	#	%
1	LORV^1^	LORV^1^	LORV^1^	Co-admin 2^4^	iNGRV-DTP^5^	oNGRV^6^	iNGRV-DTP^5^	15	21%
2	iNGRV-H^2^	LORV^1^	Co-admin 1^3^	NA	iNGRV-DTP^5^	oNGRV^6^	iNGRV-DTP^5^	7	10%
3	LORV^1^	LORV^1^	LORV^1^	LORV^1^	iNGRV-DTP^5^	oNGRV^6^	iNGRV-DTP^5^	6	8%
4	iNGRV-H^2^	LORV^1^	LORV^1^	Co-admin 2^4^	iNGRV-DTP^5^	oNGRV^6^	iNGRV-DTP^5^	6	8%
5	iNGRV-H^2^	iNGRV-M^2^	Co-admin 1^3^	NA	iNGRV-DTP^5^	iNGRV-H^2^	iNGRV-DTP^5^	4	6%
Stakeholders with Unique Preference Patterns 1–5								38	54%
Stakeholders with Unique Preference Patterns 6–25								33	46%
***Total***								***71***	***100%***

^1^Live oral rotavirus vaccine.

^2^Standalone injectable next generation rotavirus vaccine (NGRV) with high (H) and moderate (M) efficacy.

^3^LORV + iNGRV-M.

^4^LORV + iNGRV-DTP.

^5^iNGRV-DTP-Hib-HepB containing combination.

^6^Oral NGRV.

### 3.3 Overall preference pattern

Despite significant diversity of individual preference pathways, across-case analysis indicates a prominent overall trend in the sample. Whether delivered as a standalone injectable (C1) or in a more costly co-administered schedule (C3 and C4), high efficacy iNGRV was preferred by a substantial proportion of the stakeholders. When the efficacy advantage was removed from the standalone iNGRV (C2 and C6), stakeholders strongly favored oral options. However, regardless of iNGRV efficacy, preference shifts sharply towards iNGRV in all comparisons involving iNGRV-DTP, displayed prominently at C4, C5, and C7 (Fig. 1).

Preference patterns from different countries parallel these overall findings with only slight variations. The 13 stakeholders from Malawi were the most receptive to iNGRV-H (C1) and co-administered schedules (C3-C4) that offered greater protection, while unanimously rejecting less efficacious iNGRV-M (C2). In contrast, stakeholders in Sri Lanka, where rotavirus deaths and hospitalizations are low compared to other study countries, were least enthusiastic about iNGRV-H. Patterns in Ghana, Kenya, Peru, and Senegal generally reflect the all-country pattern displayed in Fig. 1.

### 3.4 Findings on three key questions

*Key Question 1: Would a standalone iNGRV be a preferred alternative to LORV if it averted more child deaths and hospitalizations, was less costly to procure, or both?* (C1-C2)

**Fig. 2:**
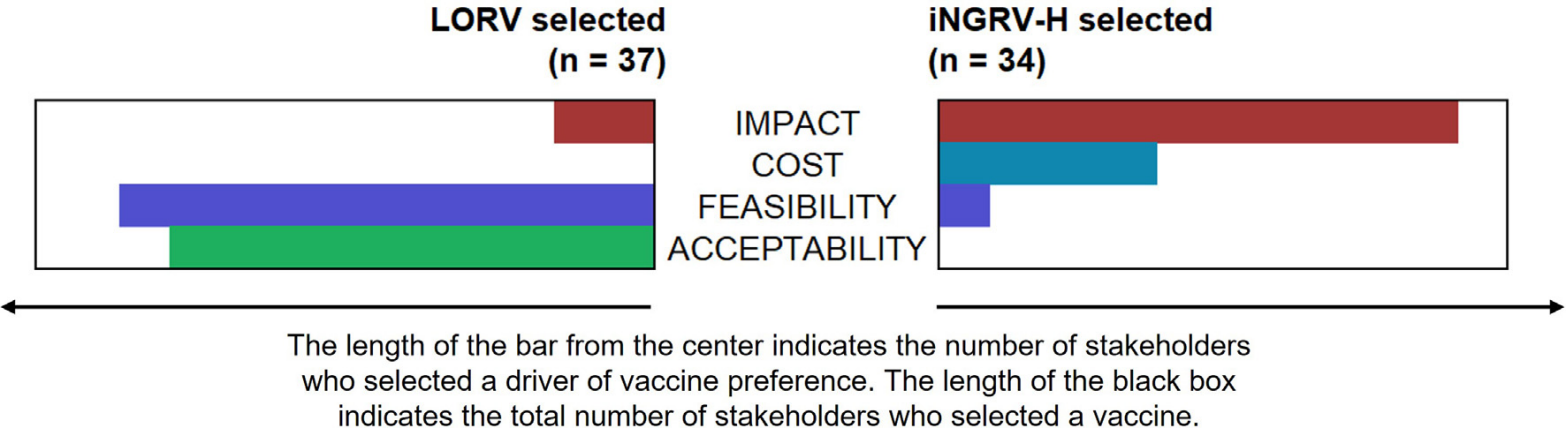
Main Reasons for Selecting LORV or iNGRV-H. LORV = live oral rotavirus vaccine; iNGRV-H = standalone injectable next generation rotavirus vaccine with high efficacy.

Of all the comparisons, selection of LORV or iNGRV-H in C1 was the most evenly split, with many stakeholders prefacing their selection as “a tough call.” Despite strongly overlapping concerns (e.g., public health impact emphasized in the quotes below), stakeholders tended to have clearly distinct reasons for selecting one option over the other in C1 (Fig. 2):

• Selected iNGRV-H: *“This one is interesting, but we have so many injectable[s] now, my concern is acceptability* … *.*… *This is the kind of thing that bothers me, each vaccine has benefits and disadvantages, but I'll choose [iNGRV]* ***mainly because of the deaths averted****.”* (S_003)
• Selected LORV: *“****What’s striking is the number of deaths [averted]. Likewise, hospitalizations****. My only concern is the number of injections. We have a problem in our country with adherence, that's my concern. If I had to make a choice, I’d stick with the [LORV].”* (S_005)

Thirty-one of 34 individuals who selected iNGRV-H indicated fewer under-five deaths and hospitalizations as a principal reason for their selection. Only 13 individuals indicated lower cost of iNGRV-H as a driver, all but one from Gavi co-financing transition phase or Gavi-ineligible countries. Among those who preferred iNGRV-H, most did so with reservations, volunteering concerns about adding injections and higher cold chain requirements. Six stakeholders, however, said they preferred delivery by injection, describing it as “more effective” by avoiding loss of vaccine from children “vomiting” oral doses or as more “hygienic” and “safe,” for example, from risk of intussusception. When the efficacy advantage of the standalone iNGRV was removed in C2 (iNGRV-M), interest in it decreased from 34 to 12 stakeholders, 11 citing lower cost of iNGRV-M as the main or sole reason (“because all the other stuff is negative”) and two repeating their preference for delivery by injection.

Stakeholders who preferred LORV over iNGRV-H tended to emphasize perceived disadvantages of the injectable as the reason for selecting LORV. In addition to programmatic burden and cost to introduce new or switch vaccines, a litany of operational considerations dominated these replies, exemplified by this stakeholder from Ghana:

*“The new vaccine is more efficacious, but****where will I store it****? And it's injectable, which will increase****waste management****problems. And my worry is that we have****injections on the schedule****at 6, 10, and 14 weeks. Another injectable will need a lot of training.****A lot of training.****How are our own staff taking it? We hear all the time, ‘The injections are too many.’ Some even get confused, and that's one, two, three more****adverse events****. At times they will even [alter the schedule****,****telling clients] to leave and come for a weekly dose or something, which is not helpful to us. So, this new option comes with its own challenges and … will****increase the cost to the program****.”* (G_001)

In contrast to these disadvantages, the “convenience” and “easy administration” of LORV were frequently cited by stakeholders who preferred it in C1 and C2. Three individuals also described a new rotavirus vaccine as “non-essential” or “not needed,” either because rotavirus was not considered as a health problem or because LORV was considered sufficiently effective.

Whether LORV or standalone iNGRV was preferred in C1-C2, with few exceptions, stakeholders were vociferous about too many injections crowding the schedule and, literally, the child’s body: “the left [limb], the right [limb], everywhere is full.” Pain experienced by the child and mothers’ fears – “the mothers are a mess” – were often mentioned as potential threats to achieving coverage targets if another injectable was added to the schedule.

In sum, as relates to Key Question 1, our findings suggest that a more effective and lower cost iNGRV may be attractive in LMICs with high rotavirus burden or concerns about financial sustainability, despite concerns about adding an injectable.

*Key Question 2: If an iNGRV is not found to be substantially more efficacious than LORVs, are there formulations in which it would be preferable to LORVs?* (C3-C5)

LORV v. Co-admin 1: C3 compares LORV to a schedule where iNGRV-M is given alongside continued delivery of LORV. We assumed this mixed schedule would improve efficacy compared to provision of LORV alone but would cost more and require more cold chain to accommodate a new injectable.

Twenty stakeholders said they preferred Co-admin 1 to LORV, most of them having also selected iNGRV-H in C1 and similarly citing improved health impact as the reason for their choice. Conversely, five stakeholders who selected LORV in both C1 and C2 opted for Co-admin 1 in C3, all citing better protection as the main reason. Fifty-one individuals who preferred LORV over Co-admin 1 expressed strong reasons for doing so:

*“Oh my god, two vaccines? Okay, there are more vaccines, more chain volume, higher cost, oral and an injectable … this is a problem. [I’d go for the] existing. [The mixed schedule] is a pan con mango (a bad combination).”* (P_001)

LORV v. Co-admin 2: C4 also involved a comparison between LORV versus a mixed LORV-iNGRV schedule (Co-admin 2). In this instance, however, LORV was co-administered with iNGRV-DTP, thereby requiring no new injections or additional cold chain storage. Like Co-admin 1, Co-admin 2 was assumed to provide substantially better protection at a moderately higher cost than the LORV alone. On the presumption that stakeholders who accepted more costly Co-admin 1 in C3, which requires a standalone injectable along with LORV, would also be amenable to Co-admin 2, which does not require an additional injectable, C4 questions were posed only with stakeholders who selected LORV in C3.

At this juncture (C4) in the comparison flow we see an important shift in preference towards iNGRV, shown in Fig. 1 by a change from predominantly gray segments at the C3 level to predominantly red/pink segments at the C4 level. Regardless of prior selections, 37/51 stakeholders selected Co-admin 2 in C4, most indicating they did so because iNGRV in a DTP-containing combination vaccine addressed one or more concerns expressed earlier in the interview. Replies like “it’s inside the penta,” “it doesn’t require a new injection,” “it’s easier for the patient,” and “convenient for the doctor” were frequently cited rationales for choosing Co-admin 2. This strong shift towards iNGRV in C4 was observed in all countries. Considering C3 and C4 preferences together, all but 14/71 stakeholders preferred a mixed LORV-iNGRV schedule over LORV alone.

**Fig. 3:**
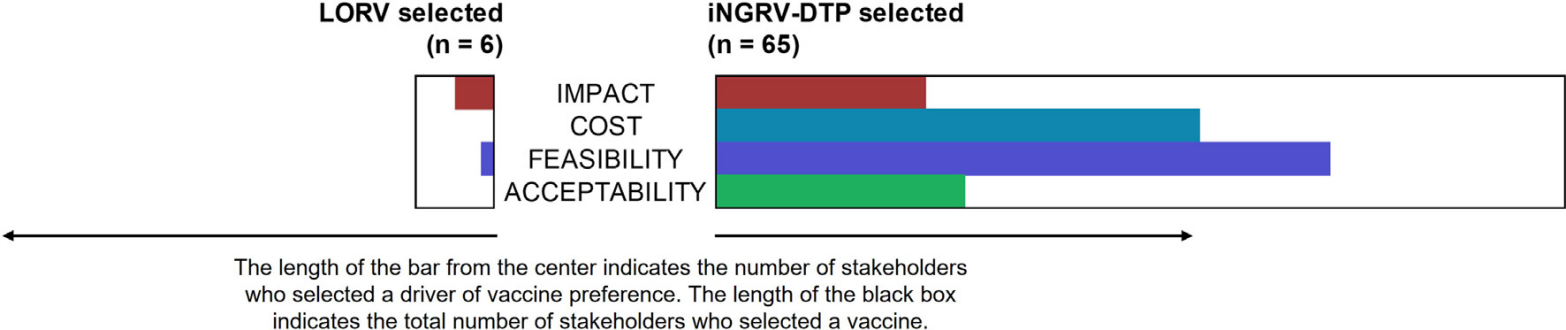
Main Reasons for Selecting LORV or iNGRV-DTP. LORV = live oral rotavirus vaccine; iNGRV-DTP = iNGRV-DTP-Hib-HepB containing combination.

LORV v. iNGRV-DTP: Continuing to explore possible interest in a less efficacious iNGRV, C5 compared LORV alone to iNGRV-DTP alone. In this instance, LORV and iNGRV-DTP were assumed to have similar efficacy, while rotavirus moiety within iNGRV-DTP was assumed to be available at substantially lower cost compared to LORV. Despite not having an efficacy advantage compared to LORV, all but six of the 71 stakeholders preferred iNGRV-DTP, emphasizing its low cost, reduced cold chain burden, avoidance of new injections, or a combination of advantages, variably expressed, e.g.: “there’s nothing new,” “it’s free,” “it removes a vaccine from the schedule.” The multiple perceived advantages of iNGRV-DTP are visually captured in Fig. 3.

Six stakeholders said they still preferred LORV. Three responses were unclear or missing, while three individuals expressed skepticism about the ability to add iNGRV to a combination vaccine or indicated greater faith in the status quo by “default.” All countries except Senegal were represented among these six individuals; three were involved in research, two held senior roles in national immunization programs, and three were serving on NITAGs.

**Fig. 4:**
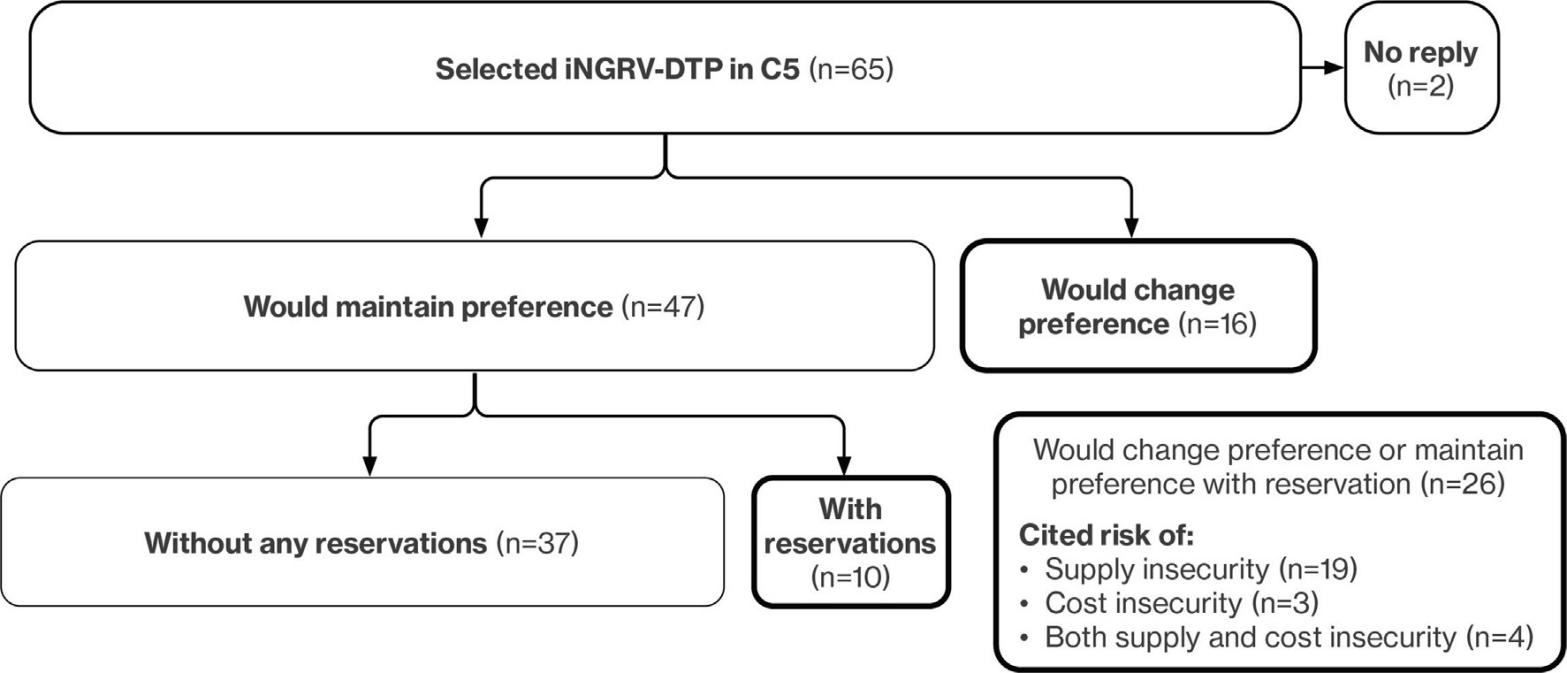
Impact on Preference for iNGRV-DTP if Available from Only One Manufacturer.

iNGRV-DTP sole supplier: To gauge concerns about the theoretical possibility of a sole supplier of iNGRV-DTP, we asked the 65 stakeholders who selected it in C5 to tell us if they would still choose iNGRV-DTP if only one manufacturer supplied it (Fig. 4). Most (n = 37) indicated that they would maintain their choice of iNGRV-DTP without hesitation (e.g., “availability must be taken into account, and it's Gavi that takes care of that” [S_004]) and ten others said they would still opt for it, but with reservations. Sixteen individuals said they would revert back to the LORV. Risk of unstable supply, high cost, or both were the main concerns which could be mitigated by a healthy marketplace:

*“Monopoly is bad, because it begins the first year they say ‘yes, yes, I give you everything,’ and then they do what they want. It is a business, and I am not going against that. But there should be competition. That is better for everyone.”* (P_001)

As relates to Key Question 2, our findings suggest a co-administered LORV + iNGRV-DTP schedule to improve efficacy may be attractive in LMICs, despite higher cost. At similar efficacy to LORV, iNGRV-DTP is highly preferred to LORV assuming there is a stable supply of the combination vaccine.

*Key Question 3: What are stakeholder preferences for and views about an oral LORV with an initial neonatal dose (oNGRV) compared to equally efficacious iNGRV options?* (C6-C7)

oNGRV v. standalone iNGRV: Paralleling C1, C6 explored perceived dis/advantages comparing equally effective standalone iNGRV versus oNGRV with an initial neonatal dose. Despite its relatively higher cost compared to iNGRV, 51/71 stakeholders preferred oNGRV for reasons similar to those given for preferring LORV in C1. Nine individuals said that early protection and/or potential for improving coverage with a neonatal dose attracted them to oNGRV. Twenty individuals preferred iNGRV over oNGRV due mainly to its provision in the routine schedule and less frequently to lower cost or perceived effectiveness of delivery by injection:

*“Neonatal vaccines create operational problems. We saw this with neonatal hepatitis. We have no coverage and don't know if the midwives inject it or not.”* (S_005)

*“The neonate might spit up. I’m not sure if I’ll be able to give all of the oral to a neonate. Injectable will be better.”* (R_001)

oNGRV v. iNGRV-DTP: Paralleling C5, C7 compares oNGRV with iNRGV-DTP, assumed to be equally effective. In C7 we again see a sharp shift in preference toward iNGRV-DTP, with 65/71 stakeholders selecting it for similar reasons as those given for preferring iNGRV-DTP in C5. Among six individuals who preferred oNGRV to the combination vaccine, four were attracted to early protection of the child and/or the possibility of improving coverage because “it’s given in the neonatal period [when mothers are] easy to catch.” Both of these reasons are illustrated clearly by this stakeholder from Peru:

*“I always wondered, why not vaccinate neonates? Even though they have maternal immunity, it's like a little help, not a replacement, for the two doses. It would be a third dose, but it makes sense to help them. Even though it's not perfect and requires two doses later, we have problems when the child doesn't receive doses at 2 or 3 months. A neonatal dose might help a little, or we may get the child easier, so if they get infected with natural rotavirus, we may attenuate it … Personally, I'm very interested in that concept, and I haven't seen it well developed so far.”* (P_013)

As relates to Key Question 3, our findings show strong interest in oNGRV, but not if an equally effective iNGRV-DTP-containing vaccine is available.

## 4 Discussion

Results from our interviews with national stakeholders provide useful nuance to prevailing assumptions about possible future demand for NGRVs in LMICs. Firstly, overwhelming preference across all countries for an iNGRV-DTP-containing vaccine over all other options, whether delivered as part a mixed schedule to improve efficacy or on its own offering no greater protection than existing LORVs, calls into question the assumption that a new rotavirus vaccine must demonstrate higher efficacy to be useful and acceptable. The multiple perceived advantages of iNGRV-DTP-containing vaccine reflect growing programmatic and financial challenges for LMICs as more vaccines are introduced [36] as well as concerns about injection fatigue and vaccine hesitancy [37].

These results are bolstered by findings on iNGRV-DTP-containing vaccine from other NGRV value proposition analyses – including strong health worker enthusiasm, greater cost effectiveness compared to other products, and potential for substantial future market share (respectively, J. Mooney, F. Debullut, and W. Hausdorff, unpublished results). They are also consistent with findings from prior research on the public health benefits of childhood combination vaccines [38]. Taken together the findings suggest that availability of a moderately (or highly) effective iNGRV delivered as part of a DTP-containing vaccine could lead both to more countries introducing rotavirus vaccine and to improved vaccination timing and coverage in individual countries. The potential public health benefit of a combination vaccine underscores the value of iNGRV licensure trials designed to demonstrate clinical non-inferiority, not only superiority, to LORV, and supports the case for increasing investments in and accelerated pathways to licensure.

Results from national stakeholders are mixed as relates to the assumption that a standalone injectable NGRV would be rejected, a view that was forcefully expressed by international experts we interviewed. On one hand, concerns about low acceptance of, adherence to, and coverage with another injectable vaccine bear out the assumption. On the other hand, almost half of the stakeholders preferred a three-dose standalone iNGRV that offers greater protection than existing LORVs, suggesting that a highly effective standalone iNGRV could be competitive in some countries, but not if an equally effective oNGRV were also available.

Our study results also differ from the assumption that a mixed LORV + iNGRV-M schedule to achieve higher efficacy would be considered by stakeholders as too complicated and/or costly to pursue. Notably, that 20/71 individuals preferred co-administration of LORV with standalone iNGRV-M to LORV delivered alone, indicates potential importance of a better performing vaccine for LMICs with high rotavirus burden. Strong preference across all countries for the co-administration of LORV with iNGRV-DTP-containing vaccine over LORV alone, despite higher cost, further indicates interest in more effective options, while also highlighting the appeal of a combination vaccine.

Finally, our findings are also somewhat mixed as concerns the general assumption that a highly effective oNGRV following a neonatal-infant schedule would be preferred over all other options. While national stakeholders expressed strong interest in oNGRV, its oral delivery and early protection advantages were not competitive against multiple perceived advantages of an equally effective combination vaccine. In hindsight, a direct comparison between a highly effective oNGRV and relatively less effective iNGRV-DTP containing vaccine would have added useful insight pertaining to this assumption.

## 5 Conclusions

While stated preferences may not directly translate into policy recommendations or actual procurements, combined with NGRV cost effectiveness analyses and demand forecasting our study results can inform future NGRV development work. Our hope is that vaccine developers, funding agencies, and policy makers utilize results presented here to improve alignment between LMIC needs and vaccine development [39], [40] and to reduce risk in new vaccine investments [41], [42].

## Funding

This work was supported by the Bill & Melinda Gates Foundation, Seattle, WA (grant number INV-007380).

## Declaration of Competing Interest

The authors declare that they have no known competing financial interests or personal relationships that could have appeared to influence the work reported in this paper.
