# Supplementary material for: National stakeholder preferences for next-generation rotavirus vaccines: Results from a six-country study
Source: Vaccine. 2022 Jan 21;40(2):370–9. doi: 10.1016/j.vaccine.2021.11.009 (PMC8767494; doi:10.1016/j.vaccine.2021.11.009)
Supplement: Supplementary data 2 [file mmc2.docx]

**NRRV F&A Study**

**National Stakeholder Interview Guide**

**Version 3: 17 October 2019**

X.0 NS Unique ID: ___ - ___ ___ - ___ ___ ___

X.1 Have you turned on the audio recording?

⃝ Yes

⃝ No

X.2 Speak into the recording:

“Starting the interview with respondent [NS Unique ID]

X.3 Date interview was conducted: DD/MM/YYYY

| **Country:** | 1 | Ghana | 5 | Peru |
| --- | --- | --- | --- | --- |
|  | 2 | Kenya | 6 | Senegal |
|  | 3 | India | 7 | Sri Lanka |
|  | 4 | Malawi |  |  |

X.4

**Introduction & Interview Initiation**

Thank you for agreeing to take part in this study.

In this interview we are thinking about the long-term horizon. As there are several different vaccine options on the horizon and each option has pros and cons, it is important for global decision-makers and vaccine developers to understand what is most valued by country stakeholders. That is why we’re interviewing you today – to understand what you value most and would prefer in future rotavirus vaccine products.

Please keep this in mind during the interview. I’m going to start by asking you a few background questions and then will ask you to compare different rotavirus vaccine product options.

**Section 1: Stakeholder Profile**

- 1. As the [title/role] in [country], how would you describe your responsibilities when it comes to vaccine policy, delivery, and/or research?

*Do not need to type the answer – audio recording will capture*

- 1. How many years have you been in your current role?

*Do not need to type the answer – audio recording will capture*

1.3 Are you a member of the NITAG or ICC or do regularly attend these meetings?

*If yes → continue to Q1.4*

*If no → skip to Q1.5*

- 1. What is your role in NITAG or ICC?

*Do not need to type the answer – audio recording will capture*

- 1. How is the decision to introduce a new vaccine made in [COUNTRY]?

*Do not need to type the answer – audio recording will capture*

- 1. What about decisions to switch to another vaccine product for the same antigen?

*Do not need to type the answer – audio recording will capture*

1.6.1 How does the process differ from that of introducing new vaccines?

*Do not need to type the answer – audio recording will capture*

1.7 From your perspective, which statement best describes the seriousness of Rotavirus as a public health problem in [COUNTRY]?

⃝ A very serious problem and one of the leading causes of child deaths

⃝ A serious problem, but not among the top causes of child deaths

⃝ Not a very serious problem compared to other childhood diseases

⃝ Don’t know.

1.7.1 What makes you respond this way?

*Do not need to type the answer – audio recording will capture*

*Sri Lanka → go to Section 2*

1.8 From your perspective, which statement best describes the impact that Rotavirus vaccine has on diarrheal disease in [COUNTRY]?

⃝ Has significantly reduced under-five mortality

⃝ Has helped reduce under-five deaths, but more needs to be done

⃝ Has not led to substantial changes in childhood diarrheal deaths

⃝ Don’t know

1.8.1 What makes you respond this way?

*Do not need to type the answer – audio recording will capture*

**Section 2: Attribute Relevance**

Thank you for your replies. Before I ask you to compare different vaccines, I want to learn a little bit about what specific vaccine attributes are most relevant to you and your current role in [country]. Please turn to page [X] of your chart where you will see Visual Aid 2.1 in blue.

**Visual Aid 2.1**

Please have a look at the list of vaccine attributes shown in the chart and let me know if you have any questions.

|  |  |  |  |  |  |  |  |
| --- | --- | --- | --- | --- | --- | --- | --- |
| Which of these seven characteristics is most important when considering introducing a new vaccine in your country? |  |  |  |  |  |  |  |
| Which of these seven characteristics is second most important when considering introducing a new vaccine in your country? |  |  |  |  |  |  |  |
| Which of these seven characteristics is third most important when considering introducing a new vaccine in your country? |  |  |  |  |  |  |  |
| Which of these seven characteristics is least important when considering introducing a new vaccine in your country? |  |  |  |  |  |  |  |

2.1 Why did you rank XXX characteristic first?

*Do not need to type the answer – audio recording will capture*

2.2 Why did you choose XXX characteristic second?

*Do not need to type the answer – audio recording will capture*

2.3 Why did you choose XXX characteristic third?

*Do not need to type the answer – audio recording will capture*

2.4 Why did you choose XXX characteristic last?

*Do not need to type the answer – audio recording will capture*

**Section 3: Vaccine Comparisons**

Thank you for your replies so far. I now want to transition to the rotavirus vaccine comparisons. There will be eight comparisons in all. For each comparison, I’m going to show you information on several attributes for the vaccines being compared and then I’ll ask you to tell me which vaccine you would prefer and why.

Before we get started on the comparisons, I want to point out some key assumptions about all of the vaccines, please turn to page [X] where you will see Visual Aid 3.0 in blue.

First, assume all vaccines are WHO prequalified and are supported by Gavi co-financing for eligible countries. We also assume a shelf-life of 24 months at 2-8°C. Also assume all vaccines have comparable safety, with good safety profiles.

Costs are adjusted to reflect [[country’s]] Gavi co-financing status in 2025. Finally, fully immunized child costs include the cost of the doses in the series only and does not include supply chain costs.

Do you have any questions about these assumptions?

Okay, thank you. Let’s get started with the first vaccine comparison.

**Visual Aid 3.0**

Please flip to page [X], you should be looking at Visual Aid 3.1 in blue, which is showing information for three existing rotavirus vaccines. Take a moment to review the information shown in the chart and let me know when you are ready or if you have any questions.

**Visual Aid 3.1**

|  | Existing Vaccine 1 | Existing Vaccine 2 | Existing Vaccine 3 |
| --- | --- | --- | --- |
| Please select which vaccine is your first choice |  |  |  |
| Please select which vaccine is your second choice |  |  |  |
| (do not ask respondent) select remaining vaccine as third choice |  |  |  |

3.1 Why did you select XXX as your first choice?

*Do not need to type the answer – audio recording will capture*

3.1.1 Even though you selected XXX as your first choice, which attribute did you find least important in this decision? **[pause]** Why did you select this attribute?

⃝ Deaths averted

⃝ Hospitalizations averted

⃝ Presentation

⃝ Route of administration & dosage

⃝ Schedule

⃝ Cold chain volume requirements

⃝ Cost per fully immunized child

Please turn to page [X], you should be looking at Visual Aid 3.2 in [color] This chart compares [selected existing vaccine] with a new injectable rotavirus vaccine that is currently being studied and may become available by 2025. The new vaccine has a greater impact on deaths and hospitalizations averted compared to the existing vaccine.

Please take a moment to review and compare the information in the chart, as the injectable vaccine differs from the oral vaccine in several other aspects. Let me know when you are ready or if you have any questions.

**Visual Aid 3.2**

3.2 Now please select which of the two vaccines you prefer.

⃝ New vaccine

⃝ Existing vaccine

3.2.1 Tell me why you have selected the vaccine you did.

*Do not need to type the answer – audio recording will capture*

3.2.2 Which statement best describes the strength of your preference for [PREFERRED VACCINE]?

⃝ Very strong

⃝ Moderately strong

⃝ I don’t have a strong preference

3.2.3 Looking at all of the attributes shown, which one did you find most important or influential to your decision and why?

⃝ Deaths averted

⃝ Hospitalizations averted

⃝ Presentation

⃝ Route of administration & dosage

⃝ Schedule

⃝ Cold chain volume requirements

⃝ Cost per fully immunized child

3.2.4 Of these same attributes, which one would describe as least important or influential to your decision and why?

⃝ Deaths averted

⃝ Hospitalizations averted

⃝ Presentation

⃝ Route of administration & dosage

⃝ Schedule

⃝ Cold chain volume requirements

⃝ Cost per fully immunized child

Please turn to page [X], you should see Visual Aid 3.3 in [color]. This chart compares [selected existing Vaccine] with a new injectable rotavirus vaccine that is currently being studied and may become available by 2025. However, in this comparison the new vaccine now has similar impact on deaths and hospitalizations averted compared to the existing vaccine.

**Visual Aid 3.3**

3.3 As you did earlier, please select which of the two vaccines you would prefer.

⃝ New Vaccine 2

⃝ Existing vaccine

3.3.1 Tell me why you have selected the vaccine you did.

*Do not need to type the answer – audio recording will capture*

3.3.2 Which statement best describes the strength of your preference for [PREFERRED VACCINE]?

⃝ Very strong

⃝ Moderately strong

⃝ I don’t have a strong preference

3.3.3 Looking at all of the attributes shown, which one did you find most important or influential to your decision and why?

⃝ Deaths averted

⃝ Hospitalizations averted

⃝ Presentation

⃝ Route of administration & dosage

⃝ Schedule

⃝ Cold chain volume requirements

⃝ Cost per fully immunized child

3.3.4 Of these same attributes, which one would describe as least important or influential to your decision and why?

⃝ Deaths averted

⃝ Hospitalizations averted

⃝ Presentation

⃝ Route of administration & dosage

⃝ Schedule

⃝ Cold chain volume requirements

⃝ Cost per fully immunized child

Please turn to page [X], you should see Visual Aid 3.4 in [color]. It is possible that significantly higher efficacy against rotavirus will only occur if BOTH the new and existing vaccines are given at the same time. I now want you to compare the existing vaccine option with a new schedule that includes both oral and injection doses.

**Visual Aid 3.4**

3.4 As you did earlier, please select which of the two vaccines you would prefer.

⃝ New Co-Admin vaccine

⃝ Existing vaccine

3.4.1 Tell me why you have selected the vaccine you did.

*Do not need to type the answer – audio recording will capture*

3.4.2 Which statement best describes the strength of your preference for [PREFERRED VACCINE]?

⃝ Very strong

⃝ Moderately strong

⃝ I don’t have a strong preference

3.4.3 Looking at all of the attributes shown, which one did you find most important or influential to your decision and why?

⃝ Deaths averted

⃝ Hospitalizations averted

⃝ Presentation

⃝ Route of administration & dosage

⃝ Schedule

⃝ Cold chain volume requirements

⃝ Cost per fully immunized child

3.4.4 Of these same attributes, which one would describe as least important or influential to your decision and why?

⃝ Deaths averted

⃝ Hospitalizations averted

⃝ Presentation

⃝ Route of administration & dosage

⃝ Schedule

⃝ Cold chain volume requirements

⃝ Cost per fully immunized child

- If LORV proceed to next comparison. If they choose co-admin, skip to Comparison 6 –

Please turn to page [X], you should see Visual Aid 3.5 in [color]. You just told me you preferred the existing vaccine over the new co-admin schedule. I now want you to compare the existing vaccine option with a new co-admin schedule where the new vaccine is provided within a DTP/penta combination that requires no additional injections.

**Visual Aid 3.5**

3.5 As you did earlier, please select which of the two vaccines you would prefer.

⃝ Co-administration of Oral + Rotavirus Vaccine in DTP/Penta combo

⃝ Existing vaccine

3.5.1 Tell me why you have selected the vaccine you did.

*Do not need to type the answer – audio recording will capture*

3.5.2 Which statement best describes the strength of your preference for [PREFERRED VACCINE]?

⃝ Very strong

⃝ Moderately strong

⃝ I don’t have a strong preference

3.5.3 Looking at all of the attributes shown, which one did you find most important or influential to your decision and why?

⃝ Deaths averted

⃝ Hospitalizations averted

⃝ Presentation

⃝ Route of administration & dosage

⃝ Schedule

⃝ Cold chain volume requirements

⃝ Cost per fully immunized child

3.5.4 Of these same attributes, which one would describe as least important or influential to your decision and why?

⃝ Deaths averted

⃝ Hospitalizations averted

⃝ Presentation

⃝ Route of administration & dosage

⃝ Schedule

⃝ Cold chain volume requirements

⃝ Cost per fully immunized child

- If Co-admin, proceed to next question. If they choose existing vaccine, skip to Comparison 6 –

3.5.6 Now consider that this new vaccine within a DTP/Penta combination vaccine is only produced by a single manufacturer. Would you still prefer the combination of Oral + Rotavirus Vaccine in DTP/Penta combo?

⃝ Yes

⃝ No

Can you please elaborate on your reply?

Please turn to page [X], you should be looking at Visual Aid 3.6 in [color]. I now want you to compare the existing vaccine with a new combo, where the new vaccine is provided within a DTP/penta combination that requires no additional injections.

**Visual Aid 3.6**

3.6 As you did earlier, please select which of the two vaccines you would prefer.

⃝ Rotavirus Vaccine in DTP/Penta combo

⃝ Existing vaccine

3.6.1 Tell me why you have selected the vaccine you did.

*Do not need to type the answer – audio recording will capture*

3.6.2 Which statement best describes the strength of your preference for [PREFERRED VACCINE]?

⃝ Very strong

⃝ Moderately strong

⃝ I don’t have a strong preference

3.6.3 Looking at all of the attributes shown, which one did you find most important or influential to your decision and why?

⃝ Deaths averted

⃝ Hospitalizations averted

⃝ Presentation

⃝ Route of administration & dosage

⃝ Schedule

⃝ Cold chain volume requirements

⃝ Cost per fully immunized child

3.6.4 Of these same attributes, which one would describe as least important or influential to your decision and why?

⃝ Deaths averted

⃝ Hospitalizations averted

⃝ Presentation

⃝ Route of administration & dosage

⃝ Schedule

⃝ Cold chain volume requirements

⃝ Cost per fully immunized child

- If new combo, proceed to next question. If they choose existing vaccine, skip to Comparison 7–

3.6.6 Now consider that this new DTP/Penta combination vaccine is only produced by a single manufacturer. Would you still prefer the new combination vaccine?

⃝ Yes

⃝ No

Can you please elaborate on your reply?

Thank you very much for all of your replies so far. We only have two more comparisons. Please turn to page [X], you should be looking at Visual Aid 3.7 in [color] This comparison looks at two new vaccines, one is an injectable that you reviewed earlier. The other involves a three-dose schedule, beginning with a neonatal dose.

**Visual Aid 3.7**

3.7 As you did earlier, please select which of the two vaccines you would prefer.

⃝ Neonatal vaccine

⃝ New vaccine 1

3.7.1 Tell me why you have selected the vaccine you did.

*Do not need to type the answer – audio recording will capture*

3.7.2 Which statement best describes the strength of your preference for [PREFERRED VACCINE]?

⃝ Very strong

⃝ Moderately strong

⃝ I don’t have a strong preference

3.7.3 Looking at all of the attributes shown, which one did you find most important or influential to your decision and why?

⃝ Deaths averted

⃝ Hospitalizations averted

⃝ Presentation

⃝ Route of administration & dosage

⃝ Schedule

⃝ Cold chain volume requirements

⃝ Cost per fully immunized child

3.7.4 Of these same attributes, which one would describe as least important or influential to your decision and why?

⃝ Deaths averted

⃝ Hospitalizations averted

⃝ Presentation

⃝ Route of administration & dosage

⃝ Schedule

⃝ Cold chain volume requirements

⃝ Cost per fully immunized child

Please turn to page [X], you should see Visual Aid 3.8 in [color]. This last comparison looks at two new vaccines, one is the injectable you reviewed earlier, a new vaccine provided within a DTP/Penta combo. The other is the same three-dose schedule, beginning with a neonatal dose.

**Visual Aid 3.8**

3.8 As you did earlier, please select which of the two vaccines you would prefer.

⃝ Neonatal Rotavirus

⃝ Rotavirus Vaccine in DTP/Penta Combo Vaccine

3.8.1 Tell me why you have selected the vaccine you did.

*Do not need to type the answer – audio recording will capture*

3.8.2 Which statement best describes the strength of your preference for [PREFERRED VACCINE]?

⃝ Very strong

⃝ Moderately strong

⃝ I don’t have a strong preference

3.8.3 Looking at all of the attributes shown, which one did you find most important or influential to your decision and why?

⃝ Deaths averted

⃝ Hospitalizations averted

⃝ Presentation

⃝ Route of administration & dosage

⃝ Schedule

⃝ Cold chain volume requirements

⃝ Cost per fully immunized child

3.8.4 Of these same attributes, which one would describe as least important or influential to your decision and why?

⃝ Deaths averted

⃝ Hospitalizations averted

⃝ Presentation

⃝ Route of administration & dosage

⃝ Schedule

⃝ Cold chain volume requirements

⃝ Cost per fully immunized child

- If new combo, proceed to next question. If they choose existing vaccine, skip to Section 4–

3.8.6 Now consider that this new DTP/Penta combination vaccine is only produced by a single manufacturer. Would you still prefer the new combination vaccine?

⃝ Yes

⃝ No

**Section 4: Interview Closure**

4.1 Other than the topics already covered in this interview, what else would say is important to country decision makers when they are deciding whether to introduce a new vaccine or switch vaccine products?

*Close the interview.*
